# Tissue degrading and remodelling molecules in giant cell arteritis

**DOI:** 10.1093/rheumatology/keae710

**Published:** 2025-01-22

**Authors:** Nobumasa Watanabe, Yuichiro Hara, Yasumasa Nishito, Mai Kounoe, Kazunari Sekiyama, Eisuke Takamasu, Takayasu Kise, Naofumi Chinen, Kota Shimada, Makoto Sugihara, Hideya Kawaji

**Affiliations:** Research Center for Genome & Medical Sciences, Tokyo Metropolitan Institute of Medical Science, Tokyo, Japan; Research Center for Genome & Medical Sciences, Tokyo Metropolitan Institute of Medical Science, Tokyo, Japan; Center for Basic Technology Research, Tokyo Metropolitan Institute of Medical Science, Tokyo, Japan; Center for Medical Research Cooperation, Tokyo Metropolitan Institute of Medical Science, Tokyo, Japan; Center for Medical Research Cooperation, Tokyo Metropolitan Institute of Medical Science, Tokyo, Japan; Department of Rheumatic Diseases, Tokyo Metropolitan Tama Medical Center, Tokyo, Japan; Department of Rheumatic Diseases, Tokyo Metropolitan Tama Medical Center, Tokyo, Japan; Department of Rheumatic Diseases, Tokyo Metropolitan Tama-Nambu Chiiki Hospital, Tokyo, Japan; Department of Rheumatic Diseases, Tokyo Metropolitan Tama Medical Center, Tokyo, Japan; Research Center for Genome & Medical Sciences, Tokyo Metropolitan Institute of Medical Science, Tokyo, Japan; Department of Rheumatic Diseases, Tokyo Metropolitan Tama-Hokubu Medical Center, Tokyo, Japan; Research Center for Genome & Medical Sciences, Tokyo Metropolitan Institute of Medical Science, Tokyo, Japan

**Keywords:** giant cell arteritis, multinucleated giant cells, granuloma, molecular marker, osteoclast

## Abstract

**Objectives:**

GCA is a granulomatous vasculitis affecting large vessels, leading to intimal occlusion accompanied by the accumulation of myofibroblasts. Histopathologically, GCA is characterized by destruction of the tunica media and hypertrophy of the intima with invasion of activated CD4+ T cells, macrophages and multinucleated giant cells (MNGCs). Despite these well-defined histopathological features, the molecular pathology of GCA has largely remained elusive. We aimed to characterize the pathologic features of GCA at the molecular level.

**Methods:**

To identify key molecules involved in GCA pathogenesis, we conducted genome-wide gene expression profiling on arterial lesions obtained through temporal artery biopsy of 16 patients who had not received any prior treatment. The resulting data were examined to reveal specific pathways and genes, and some of the molecules were followed up by immunohistochemistry.

**Results:**

Our analysis revealed a unique gene expression pattern in GCA lesions, including enrichment of immune cells and phagocytic pathways related to microglia and osteoclasts. Subsequent immunohistochemistry analysis identified the presence of MMP12 (macrophage elastase), HLA-DRA, and phagocytosis- and osteoclast-associated molecules in infiltrating macrophages and MNGCs. Additionally, we discovered LRRC15-expressing cells in the tunica intima, suggesting a myofibroblast subpopulation that suppresses cytotoxic CD8+ T cells. These molecules were upregulated in other granulomatous diseases affecting not only arteries but also lymph nodes.

**Conclusion:**

Our study revealed novel molecules associated with the pathological features of GCA, providing a foundation for better understanding of GCA pathogenesis and development of targeted therapeutic strategies.

Rheumatology key messages

- RNA profiling of temporal artery biopsy identified osteoclast-like signature to GCA.
- Molecules contributing to tissue degradation, immune responses and myofibroblast accumulation were confirmed with immunohistochemistry.
- The signature molecules identified are shared among other granulomatous diseases.

## Introduction

Vasculitis syndromes, inflammatory disorders of blood vessels, are classified according to the size of the affected vessels [1]. GCA and Takayasu arteritis (TAK), which affect large vessels, are both granulomatous arteritis [2]. Whereas TAK typically manifests as inflammation of the aorta in young women, GCA affects the cranial and extracranial arteries in men and women older than 50 years [1, 2]. GCA is well characterized histopathologically, but our understanding of its molecular pathology and precise aetiology remains limited.

Occlusion of the temporal, carotid and (occasionally) coronary arteries can lead to blindness, and cerebral and myocardial infarction [2]. Underlying pathological features are intimal hyperplasia, destruction of the tunica media, and immune responses associated with CD4+ T cells. Myofibroblasts accumulate in the tunica intima, causing vessel occlusion [3, 4]. Macrophage infiltration, the widespread appearance of multinucleated giant cells (MNGCs), and immune responses including CD4+ T cell activation occur in the tunica media and adventitia of affected vessels [2, 3]. Histological examinations, often coupled with the detection of specific cells or molecules have provided a fundamental understanding of GCA pathology. Recently, omics studies investigating the DNA methylation [5], microRNAs [6] and spatial transcriptomics [7] have refined our molecular understanding of GCA. However, transcriptome data derived from the treatment-naïve arteries, which is essential to understand GCA pathogenesis, remains to be explored.

MNGCs form under both physiologic and pathologic conditions [8, 9]. Physiologic MNGCs include the osteoclasts, considered to arise from macrophages, in bone homeostasis [10]. Pathologic MNGCs, including Langhans giant cells and foreign body giant cells, are found under chronic inflammatory conditions, such as granulomatous lesions [8, 9]. They often populate the granulomas that contain epithelioid cells and M2-deviated macrophages in tuberculosis (TB) or sarcoidosis (SA) [11]. Although the morphologic features of pathologic MNGCs have been well characterized in diverse granuloma-associated inflammations, their contributions to pathogenesis are poorly understood.

In this study, we aimed to characterize the pathologic features of GCA at the molecular level. Genome-wide gene expression profiles of primary lesions obtained through temporal artery biopsy (TAB) identified a distinguishing pattern of gene expressions. Subsequent analysis expanded the list of distinctive molecules in infiltrating macrophages and MNGCs, which implicated their diverse contributions to pathogenesis. Comparison of several granulomatous diseases suggested a shared molecular basis across disorders affecting not only arteries but also lymph nodes.

## Methods

### Sex as a biological variant

Our study examined male and female subjects, and similar observations are found for both sexes.

### Patients

This study was conducted in accordance with the 1964 Declaration of Helsinki. All participants provided written informed consent. The study design was approved by the Ethics Committees of Tokyo Metropolitan Institute of Medical Science (no. 20–51), Tokyo Metropolitan Tama-Hokubu Medical Center (no. 3–20), Tokyo Metropolitan Tama Medical Center (no. 206) and Tokyo Metropolitan Tama-Nambu Chiiki Hospital (no. 30–04). Patients who were suspected as having GCA and subsequently underwent TAB as part of the diagnostic procedure were recruited. The clinical and demographic characteristics are summarized in Supplementary Tables S1 and S2, available at *Rheumatology* online.

### RNA extraction and microarray analysis

The remaining small blocks (<10 nm) of the specimen used for diagnosis, stored at 4°C in All-protect Solution (Qiagen, Hilden, NRW, Germany) in less than 1 year, were homogenized in Trizol by Tissue-Lyzer (Qiagen, Hilden, NRW, Germany). RNA was extracted by using RNeasy (Qiagen, Hilden, NRW, Germany), and its quality was assessed with 2100 bioanalyzer (Agilent Technologies, Santa Clara, CA, USA). Cy3-labelled cRNA was prepared using Low Input Quick Amp Labelling Kit (Agilent Technologies, Santa Clara, CA, USA), and hybridized to Agilent SurePrint G3 Human GE Array (version 3.0, 8 × 60K; Agilent Technologies, Santa Clara, CA, USA). SureScan Microarray Scanner G2600D (Agilent Technologies, Santa Clara, CA, USA) and Feature Extraction software (version 12.1.1.1, Agilent Technologies, Santa Clara, CA, USA) were used for image scanning and signal intensity quantification.

### Expression quantification and sample classification

Signal intensities were processed by Limma v3.42.0 [12] to quantify gene expression with background correction and normalization. Probes without positive signals or not linked to protein-coding genes were excluded. Principal component analysis (PCA), k-means clustering (k = 2) and hierarchical clustering were performed by precomp, k-means and hclust (ward.D2 method with Euclid distance) in R (Supplementary Fig. S1, available at *Rheumatology* online).

### Differentially expressed genes and cell-type enrichment

Differential analysis was performed by Limma, where log_2_FC >1 and FDR (false-discovery rate) <0.05 were considered as significant. Comparing typical GCA patients with non-/atypical cases revealed 3745 upregulated and 3706 downregulated probes (Supplementary Fig. S2A, available at *Rheumatology* online). Other comparisons are shown in Supplementary Fig. S2B–D, available at *Rheumatology* online. We selected the probe with the highest average signal across 16 samples for quantifying gene expressions [13]. This identified 2858 upregulated and 2744 downregulated differentially expressed genes (DEGs) in typical GCA (Supplementary Fig. S2D and Table S3, available at *Rheumatology* online). Supplementary Table S4, available at *Rheumatology* online, lists the diseases compared with GCA and their data sources. Only for the analysis with TAK, the top 300 genes were considered as significant. Enrichment of biological pathways was assessed by g:Profiler (accessed in May 2023) [14]. Enrichment scores of 64 immune and stromal cell types within the tissue profiles were inferred by xCells 1.1.0 [15].

### Histology and immunohistochemistry

Formalin-fixed paraffin-embedded tissue sections (4 µm) were stained with haematoxylin and eosin, Elastica van Gieson and Picrosirius red for histologic analysis. Sections were deparaffinized and rehydrated, and underwent heat-induced epitope retrieval in 10 mM citrate buffer (pH 6), 10 mM Tris-EDTA buffer (pH 9) or 1 mM EDTA (pH 8), depending on the antibody (Supplementary Table S5, available at *Rheumatology* online). Endogenous peroxidase activity was blocked with 0.5% H_2_O_2_ in 10 mM PBS (pH 7.4) for 30 min. Sections were washed in PBS with 0.03% Triton X-100 (PBST), blocked with 5% normal bovine serum (NBS) in PBS (NBS^+^PBS) for 20 min, and incubated overnight at 4°C with primary antibodies diluted in NBS^+^PBS. Slides were incubated with biotinylated secondary antibodies (dilution 1:1000) for 2 h at room temperature and visualized using a standard peroxidase-based method (Vectastain Elite, ABC kit, Vector Laboratories, Newark, CA, United States) and 3,3′-diaminobenzadine as the chromogen, followed by haematoxylin counterstaining. Sections were washed for 10 min in PBST three times. A polarizing microscope (model BX53, Olympus, Hachioji-shi, Tokyo, Japan) was used for tissues stained with Picrosirius red, and an inverted Keyence BZ-X810 microscope (Keyence, Osaka-shi, Osaka, Japan) and a CFI Plan Apo 20×/0.75 objective (Nikon, Shinagawa-ku, Tokyo, Japan) or CFI Plan Apo 40×/0.80 objective (Nikon, Shinagawa-ku, Tokyo, Japan) were used for the rest.

## Results

### Histologic features of biopsy tissue from patients with suspected GCA

We enrolled 16 patients suspected of having GCA, who had not received any treatment for their symptoms, and underwent TAB for diagnosis (Supplementary Table S1, available at *Rheumatology* online). Of them, 10 were diagnosed as GCA based on the ACR diagnostic criteria [16] and the remaining six were not. The proportion of female patients diagnosed with GCA (50%) was higher compared with non-GCA patients (17%), despite a similar median age at diagnosis. Histopathology revealed minimal immune response in two of the GCA patients, G2 and G4 (Supplementary Fig. S3A, available at *Rheumatology* online). We refer them as atypical GCA samples and the others as typical GCA hereafter. We obtained portions of the residual biopsy specimens through standard procedures.

**Figure 1.**
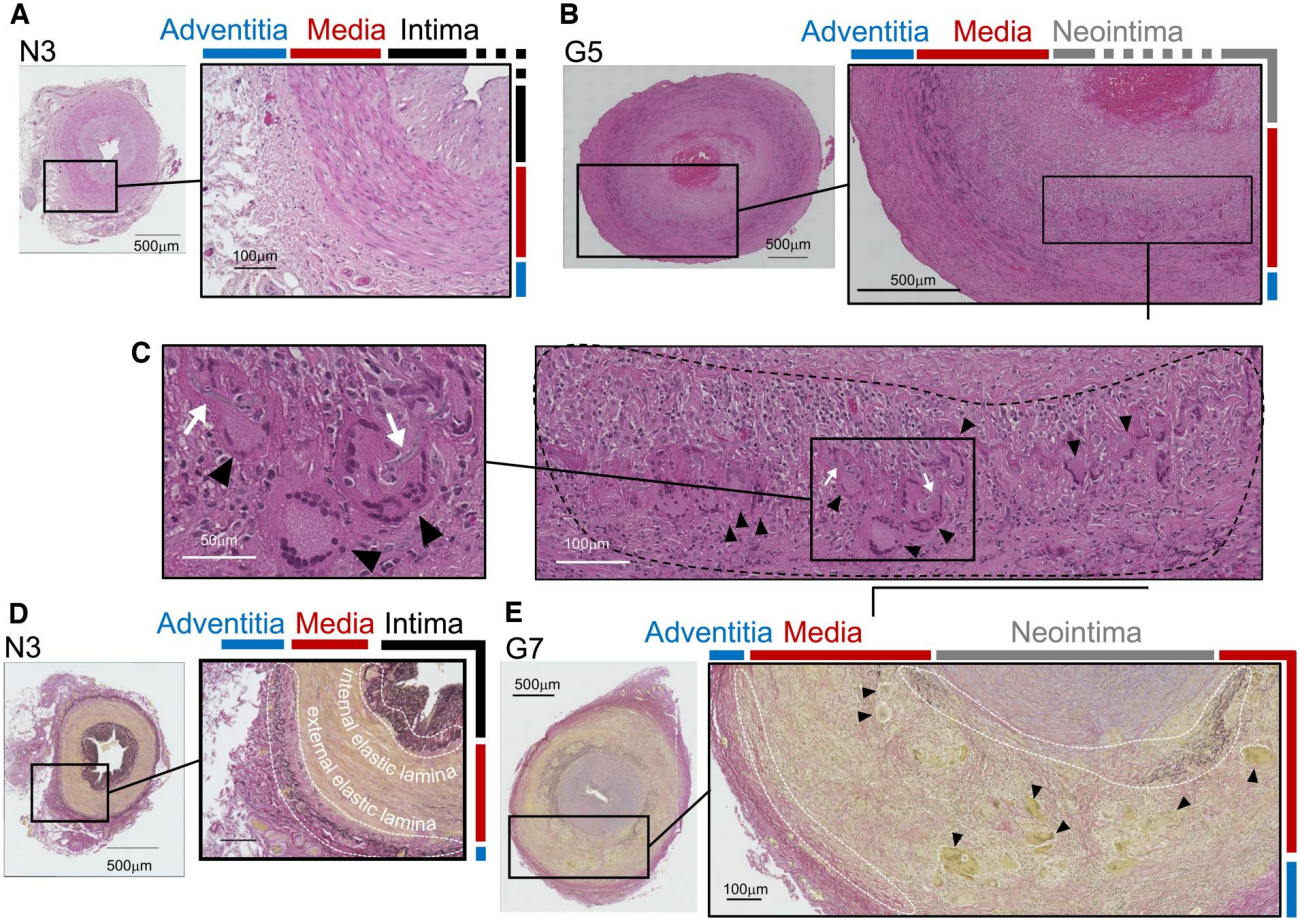
Histology of the arteries in GCA and non-GCA samples that underwent transcriptome analysis. Representative images of the biopsy tissue derived from the same participants. (**A**) Haematoxylin and eosin staining of the entire arterial cross-section of non-GCA participant N#, indicating a well organized structure of tunica intima, tunica media and tunica adventitia. (**B**) Haematoxylin and eosin staining for GCA patient G5. (**C**) Magnified images of the haematoxylin and eosinstaining in (B). Black arrowheads and white arrows indicate multinucleated giant cells (MNGCs) and the bundle-like struture within the MNGCs. A black dashed circle indicates the area with infiltration on immune cells and appearance of MNGCs. (**D**) Elastica van Gieson staining of the same biopsy tissue of N3. Elastin and collagen fibres are stained black and pink, respectively. White dashed circles indicate the areas of the internal and external elastic lamina of non-GCA artery. (**E**) Elastica van Gieson staining for GCA patient G7. White dashed circles indicate disorganization or disappearance of the elastic lamina. Black arrowheads indicate MNGCs

Haematoxylin and eosin staining indicated well-defined structure of the tunica media in non-GCA cases, mainly composed of smooth muscle cells (Fig. 1A, N3). In contrast, significant infiltration by mononuclear cells (marked by black dotted circles) including MNGCs (arrowhead) was observed in the tunica media of typical GCA cases (Fig. 1A, G1, G5, G7; Supplementary Fig. S3A, available at *Rheumatology* online).

Elastica van Gieson staining indicated elastin fibre, a primary component of the elastic lamina, at the internal and external boundaries of the tunica medial in non-GCA (Fig. 1B, white dotted circle). Conversely, the fibre structures were less evident, fragmented or absent in typical GCA cases, accompanied by appearance of MNGCs (G1, G5, G7 of Fig. 1B; Supplementary Fig. S3B, available at *Rheumatology* online).

Picrosirius red staining highlighted collagen fibres, another primary component of the elastic lamina, at the boundaries of the tunica media. A comparison between GCA and non-GCA cases indicated complete loss of the internal elastic lamina (Supplementary Fig. S3C, upper panel, available at *Rheumatology* online). Examination of collagen types with polarizing microscopy of the Picrosirius red staining showed disappearance of type I collagen (seen in yellow under polarized light) associated with the internal elastic lamina (Supplementary Fig. S3C, lower panel, available at *Rheumatology* online). These results confirmed the distinctive morphological features of typical GCA cases, compared with atypical and non-GCA cases.

### Gene expression signatures of GCA

**Figure 2.**
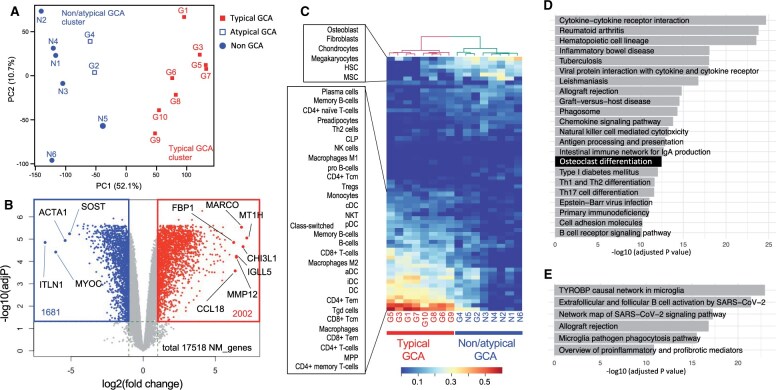
Transcriptome analysis of temporal arterial biopsies (TABs). (**A**) Principal component (PC) analysis of the transcriptomes derived from GCA and non-GCA TAB specimens. Each dot represents a single specimen, which is categorized as typical, atypical or non-GCA. The result of k-means clustering (k = 2) was shown by colour (blue and red). (**B**) Volcano plot comparing differentially expressed genes (DEGs) between the typical GCA cluster and non-GCA/atypical GCA cluster. Gray, not significantly differentially expressed; red, upregulated in typical GCA [log2FC >1 and false-discovery rate (FDR) <0.05]; blue, downregulated in typical GCA (log2FC <−1 and FDR <0.05). *P*-values were calculated by FDR-corrected empirical Bayes moderated T statistics in the R package Limma. Only protein-coding genes are shown. The positions and names of several representative genes are indicated in black. *ACTA1*, actin alpha 1, skeletal muscle; *CCL18*, C-C motif chemokine ligand 18; *CHI3L1*, chitinase 3-like 1; *FBP1*, fructose-bisphosphatase 1; *IGLL5,* immunoglobulin lambda-like polypeptide 5; *ITLN*, intelectin 1; *MARCO*, macrophage receptor with collagenous structure; *MMP12*, matrix metallopeptidase 12; *MT1H*, metallothionein 1H; *MYOC*, myocilin; *SOST*, sclerostin. (**C**) Estimation of the cellular composition of TABs, with a heatmap showing hierarchical clustering of the specimens. The xCell platform was used for the analysis. (**D**) Enrichment analysis of the Kyoto Encyclopedia of Genes and Genomes biological pathway by using the g:Profiler online tool. Upregulated DEGs (2858 genes; FDR, <0.05 and log2FC >1) were used for the analysis, and significant pathways (adjusted *P*, <1 × 10e-10) are shown. (**E**) The WikiPathways database was used to analyse the same upregulated DEGs as in D. An adjusted *P*-value of <1 × 10e-10 was used as the threshold

We performed genome-wide gene expression profiling of the obtained specimens by using a microarray platform. PCA of the expression profiles revealed clear segregation of the patients with typical GCA from the others, even for the first principal component (Fig. 2A). Notably, the cases with atypical GCA (G2 and G4) belong to the group of non-GCA patients, which is consistent with their morphologic features. The clustering based on the expression profiles identified two patient groups corresponding to ‘typical GCA’ and the others, either by k-means clustering (*k *=* *2) or by hierarchical clustering (Fig. 2A, Supplementary Fig. S1, available at *Rheumatology* online).

We explored DEGs between the typical GCA and non-/atypical GCA groups (see Methods) and found 2002 upregulated and 1681 downregulated protein-coding genes in typical GCA (log_2_FC >1 and FDR <0.05) (Fig. 2B). Given that three cases (G2, G4 and N5) exhibited intermediate molecular profiles, we performed a differential analysis excluding these cases (Supplementary Fig. S2C, available at *Rheumatology* online). We found a substantial overlap in the DEGs (Supplementary Fig. S2D, available at *Rheumatology* online), indicating that our findings are largely unaffected by the intermediate cases. By manual inspection of the top 50 genes upregulated in typical GCA (Supplementary Table S6, available at *Rheumatology* online), we noted genes that play a central role in adaptive immune responses. Macrophage-lineage genes were also evident, including *CHI3L1*, *MARCO* and *FBP1*. We examined the enriched cell types by xCells, which infers the contributions of 64 immune and stromal cell types in expression profiles of bulk samples [15]. The analysis highlighted substantial contributions of CD4+ T cells, CD8+ T cells, macrophages and multipotent progenitors (Fig. 2C). We evaluated the molecular pathways according to KEGG [18] and WikiPathways [19] (Fig. 2D and E). We found significant enrichment of immune-related pathways, particularly those related to microglia, a specialized phagocyte in the macrophage lineage in the CNS [20]. These pathways included the TYROBP causal network in microglia and those associated with microglial pathogen phagocytosis and phagosomes. We also found enrichment of the osteoclast differentiation pathway. Osteoclasts are physiologic MNGCs with a specialized function in bone resorption, and the analyses revealed upregulation of the well-known osteoclast markers *ACP5*, *MMP9* and *DCSTAMP* (Supplementary Table S6, available at *Rheumatology* online). Our gene expression profiling of temporal artery lesions demonstrated distinguishing gene expression signatures in typical GCA and implied potential molecules associated with pathogenesis, such as phagocytosis and tissue destruction in addition to inflammatory responses.

### Osteoclast-associated molecules found in MNGCs and infiltrating macrophages

**Figure 3.**
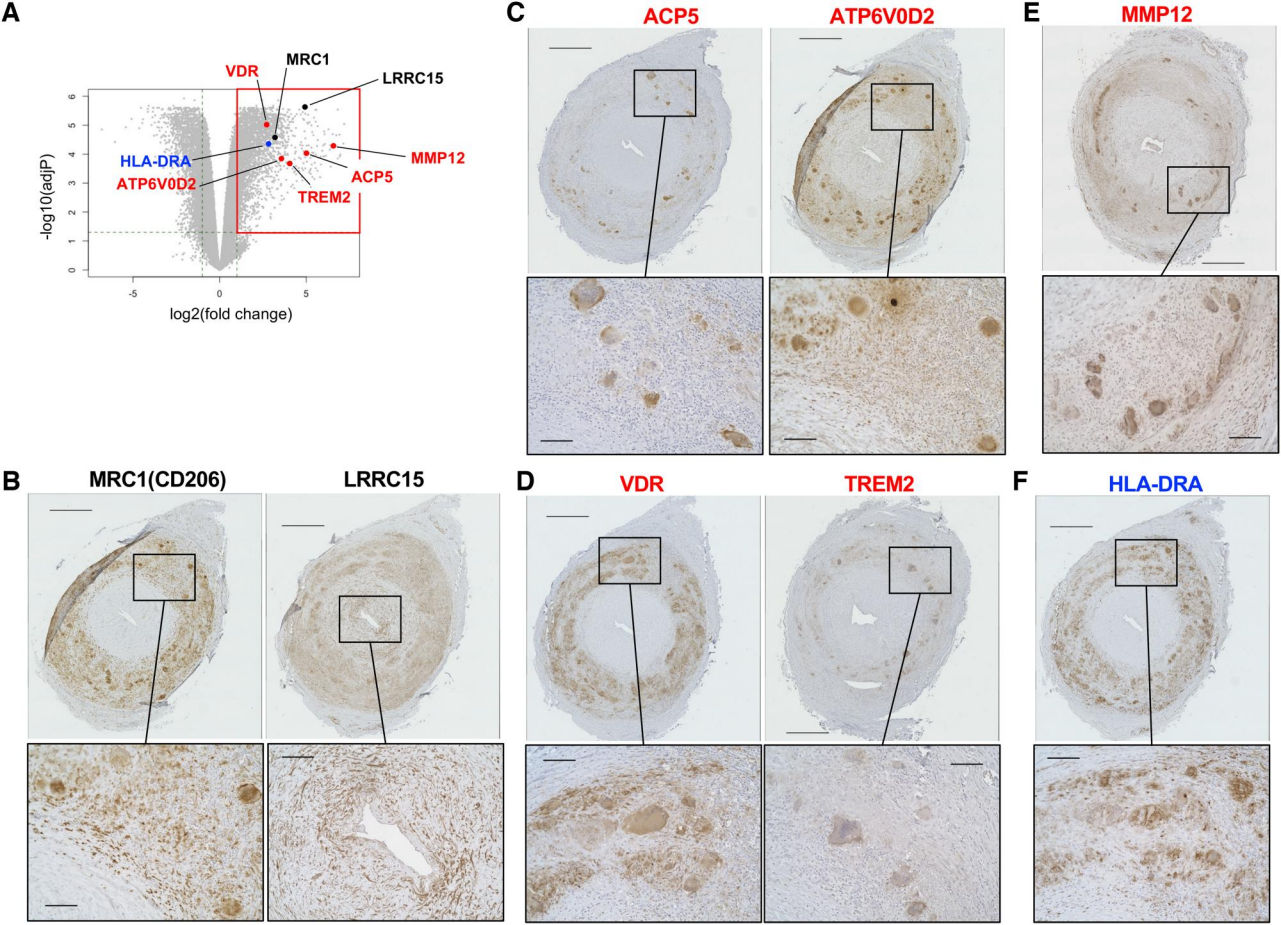
Immunohistochemical validation of differentially expressed gene expression. (**A**) The upregulated genes examined by immunohistochemistry overlaid on the volcano plot. *ACP5*, acid phosphatase 5, tartrate resistant; *ATP6V0D2*, ATPase H+ transporting V0 subunit d2; *HLA-DRA*, major histocompatibility complex, class II, DR alpha; *LRRC15*, leucine-rich repeat-containing 15; *MRC1*, mannose receptor C-type 1; *MMP12*, matrix metallopeptidase 12; *TREM2*, triggering receptor expressed on myeloid cells 2; *VDR*, vitamin D receptor. Black, genes associated with intimal hyperplasia (i.e. *LRRC15*, *MRC1*); blue, gene associated with the CD4+ T cell immune response (*HLA-DRA*); red, genes associated with destruction of the tunica media (i.e. *ACP5*, *ATP6V0D2*, *MMP12*, *TREM2*, *VDR*). (B–F) Formalin-fixed paraffin-embedded specimens of temporal arterial biopsies from a representative patient with typical GCA (G7) stained with antibodies to (**B**) MRC1 and LRRC15, (**C**) APC5 and ATP6V0D2, (**D**) VDR and TREM2, (**E**) MMP12 and (**F**) HLA-DRA. The experimental conditions, buffer pH used for heat-induced epitope retrieval, and antibody concentrations are summarized in Supplementary Table S5, available at *Rheumatology* online. In each panel, the lower images are magnifications of the boxed area in the upper image. Scale bars: upper images, 500 μm; lower images, 100 μm

We performed immunohistochemistry to examine cells expressing the molecules indicated in the gene expression analysis (Fig. 3, Supplementary Fig. S4, available at *Rheumatology* online). We aimed to confirm the features of the infiltrating macrophages and accumulated myofibroblasts. MRC1 (CD206) is an established marker for tissue-remodelling macrophages [21], and LRRC15 is a marker of myofibroblasts that suppress cytotoxic CD8+ T cells [22]. The MNGCs and surrounding macrophages in the tunica media strongly expressed MRC1 (Fig. 3B), as shown before [23]. In addition, the LRRC15 expression in the myofibroblasts of the tunica intima (Fig. 3B) confirmed the presence of a myofibroblast subpopulation under the pathogenic condition of GCA.

We next selected osteoclast-associated molecules, ACP5 and ATP6V0D2, from a well-curated list of osteoclast markers [24]. We found that ACP5, a marker of authentic osteoclasts, was expressed exclusively in the MNGCs of GCA (Fig. 3C). Similarly, ATP6V0D2 was expressed in MNGCs, with a faint signal in the macrophages of the tunica media (Fig. 3C). We examined two additional molecules related to osteoclasts: vitamin D receptor (VDR) and TREM2. Immune cells, including T cells, B cells as well as macrophage-lineage cells, express VDR and exhibit an immune-regulatory response to vitamin D [25]. VDR expressed in osteoclasts has been suggested to play a primary role in bone homeostasis [26]. TREM2, shown to play an important role in osteoclast differentiation in concert with the adaptor molecule called TYROBP [20]. We detected VDR signals in the MNGCs and infiltrating macrophages of GCA cases but not in those of non-GCA (Fig. 3D and Supplementary Figs S5 and S6, available at *Rheumatology* online). TREM2 was expressed in MNGCs (Fig. 3D). These findings revealed that the osteoclast-associated molecules are produced by the pathologic MNGCs in GCA.

### Functional molecules in tissue degradation and immune stimulation

We extended the immunochemical analysis to two additional molecules that have pathologic implications. MMP12 is a macrophage elastase that degrades extracellular matrix fibres, in particular elastin [27], and that is involved with granuloma formation [28]; *MMP12* was one of the top five upregulated genes in typical GCA (Supplementary Table S6, available at *Rheumatology* online). The fold change for *MMP12* (Fig. 3A) was substantially higher than that for *MMP9* (Supplementary Table S6, available at *Rheumatology* online), which is consistent with the previous report [29]. The MNGCs of GCA clearly stained for MMP12 (Fig. 3E).

The HLA-DR molecule is indispensable for the immune-stimulatory function of antigen-presenting cells, including dendritic cells, induced (activated) dendritic cells as well as macrophages (especially, M1 macrophages) [21, 30]. We noted HLA-DRA signals in the MNGCs as well as the infiltrating macrophages in the tunica media of GCA arteries (Fig. 3F), consistent with the previous report showing the infiltration of HLA-DR+ cells [31, 32]. These results highlight additional functions of the MNGCs as contributing to the degradation of the extracellular matrix and induction of immune responses.

### Shared molecular basis across granulomatous diseases

**Figure 4.**
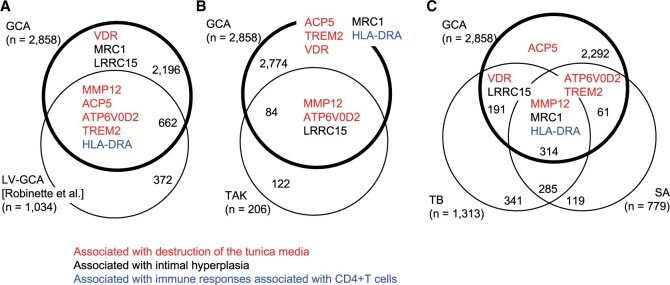
Comparative transcriptome analysis between GCA and other inflammatory disorders. (**A**) Venn diagram showing the upregulated differentially expressed genes (DEGs) common between GCA (this study) and inflammatory aortic aneurysm [diagnosed as large-vessel GCA (LV-GCA) (36)]. (**B**) Venn diagram showing the upregulated DEGs common between GCA (this study) and aortic tissue of Takayasu arteritis [TAK (37)] (**C**) Venn diagram showing the upregulated DEGs common among GCA (this study) and mediastinal lymph node tissues from patients with tuberculosis [TB (38)] or sarcoidosis [SA (38)]. Genes whose expression was validated through immunohistochemistry (Fig. 3) are shown. Colour-coding and gene acronyms are as described in Fig. 3

We asked whether any of the molecular features of GCA that we identified were consistent with those previously reported. We compared the upregulated genes in our typical GCA cases with those in the inflamed aortic tissues of large-vessel type GCA [33], a subclass of GCA (Fig. 4A). This analysis disclosed a significant overlap between the two sets (*P *=* *1.8e-281, Fisher’s exact test) that included five of the eight molecules revealed through immunohistochemistry. In particular, four of them—*ACP5*, *ATP6V0D2*, *MMP12* and *TREM2*—contribute to the destruction of the tunica media, and the remaining one—*HLA-DRA*—causes immune stimulation. These molecules in the MNGCs and infiltrating macrophages likely form a shared basis of GCA, irrespective of whether the temporal artery or aorta are affected. We also found that some of the DEGs, such as *MARCO*, *CCL18*, *CXCL9* and *SPP1*, are upregulated in another study of GCA with 770 immune-related genes [17], indicating involvement of these immune-related genes in the GCA pathogenesis.

We next focused on TAK [34], another granulomatous arteritis affecting large vessels. We found a significant overlap between the sets of upregulated genes (*P *=* *5.7e-14, Fisher’s exact test) that included three genes identified by immunohistochemistry (Fig. 4B). Further extending the comparison to lymphoid granulomas likewise revealed significant overlaps between GCA and TB (*P *=* *1.2e-80) and SA (*P *=* *2.2e-62) [35] (Fig. 4C). These overlaps indicate that the granulomatous diseases of TAK, SA, TB and GCA share molecular features associated with intimal hyperplasia and the destruction of the tunica media.

## Discussion

In this study, we investigated the arterial lesions of GCA obtained through TAB (Fig. 1) and determined their distinguishing gene expression patterns (Fig. 2). The tunica media contains infiltrating macrophages and MNGCs, which presumably originate from the macrophages. We revealed that these macrophage-lineage cells express a diverse list of molecules relevant to the pathogenesis: MMP12, important in degrading elastin [27]; ACP5 and ATP6V0D2, bone-resorbing molecules in osteoclasts [24]; VDR and TREM2, involved in osteoclastgenesis [20, 26] as well as phagocytosis [36, 37]; MRC1, a functional marker of tissue-remodelling macrophages, which induce fibrosis [38]; and HLA-DRA, necessary for antigen-presentation to CD4+ T cells [39] (Fig. 3). The myofibroblast-like cells prominently expressed LRRC15. These molecules may represent promising therapeutic targets. We found these molecules in other granulomatous diseases that affect not only arteries but also lymph nodes (Fig. 4), implying a shared molecular basis in their pathogenesis.

A key strength of our study is the use of molecular profiles from drug-naïve GCA lesions, which distinguishes our study from existing molecular studies of GCA. Despite the strengths, our study has several limitations. First, the diversity of cases analysed, including both typical and atypical GCA presentations and other forms of vasculitis (Supplementary Table S1, available at *Rheumatology* online), may affect the sensitivity of the differential analysis, although the list of DEGs appears largely robust (Supplementary Fig. S2D, available at *Rheumatology* online). Second, while treatment-naïve samples are rare, the small cohort size (*n* = 16) limit the power of statistical analysis. The expression levels of the novel marker genes in the typical GCA cases (*n* = 8) appear to be associated with several clinical parameters (Supplementary Fig. S7, available at *Rheumatology* online), but any of these differences did not reach statistical significance. Third, our analysis excluded non-coding RNA data, which could offer additional insights into GCA pathogenesis. Finally, the findings lack validation in larger, independent cohorts or in other vascular territories affected by GCA, which would enhance their broader applicability.

The increased physical strength and elasticity of large arteries are primarily due to the smooth muscle cells, collagen and elastin fibres of the tunica media [40]. This structure is damaged with the infiltration of macrophages and MNGCs in granulomatous arteritis like GCA. Previously identified molecules include MMP9, a matrix metalloproteinase involved in the degrading collagen and elastic fibres [41–44], and MRC1 which is induced by GM-CSF and may contribute to inflammatory and tissue-remodelling responses [23, 45, 46]. However, they did not explain all of the damage in the granulomatous lesions. We revealed that MMP12, a macrophage elastase, is present both in the infiltrating macrophages and the MNGCs (Fig. 3). Considering the other MMP molecules present in GCA lesions, MMP9 and MMP2 also have elastinolytic activity [43]; this finding suggests that elastic fibres are yet another target of direct destruction.

We found that the MNGCs highly express the osteoclast markers ACP5 and ATP6V0D2 (Fig. 3), which are required for bone homeostasis, including the dissolution of hydroxyapatite [8]. ATP6V0D2 is a component of the proton pump that induces acidic conditions in the regions around osteoclasts, and ACP5 is a phosphatase in acidic conditions, although its substrate remains unclear [8]. The presence of these osteoclast-associated molecules indicated that activation of the macrophages and MNGCs alters their environments. VDR is a nuclear receptor that uses the active form of vitamin D3 to alter gene expression, such as upregulation of CRIg (VSIG4), to induce phagocytosis [36]. TREM2 is a receptor expressed in both osteoclasts and microglia, and it mediates the phagocytic clearance of apoptotic cell debris [20]. The presence of VDR and TREM2 in the MNGCs, with increased expression of *VSIG4* in GCA (see Supplementary Table S3, available at *Rheumatology* online) indicates the activation of a phagocytic regulatory program.

The tunica intima, an adjacent layer to the tunica media, consists of endothelium and an internal elastic lamina in healthy conditions but becomes hypertrophied and forms a neointima, leading to vascular stenosis in GCA. Our data showed that neointimal formation is accompanied by the accumulation of myofibroblast-like cells (Fig. 1). A specific subpopulation of cancer-associated fibroblasts, defined by LRRC15 expression, suppresses cytotoxic CD8+ T cells [22]. Our data indicated that the myofibroblast-like cells in the neointima express LRRC15 (Fig. 3), suggesting immune-suppression in the hypertrophied tunica intima. This is likely the consequence of interaction with components of the tunica media, in particular with MRC1-expressing macrophage-lineage cells, given that MRC1-positive macrophages promote myofibroblast differentiation and proliferation [47].

The activation of CD4+ T cells is another pathologic feature of GCA. HLA-DRA functions in antigen-presentation to CD4+ T cells, which is a prerequisite for their activation, and HLADRB04 is genetically associated with GCA [3]. Because vascular-resident dendritic cells stimulate CD4+ T cells through MHC class II, dendritic cells are considered to contribute to the initial phase of GCA pathogenesis [30]. The infiltration of CD4+ T cells as well as CD8+ T cells in GCA is supported by our data, where substantial activation of CD4+ T cells were estimated (Fig. 2C). The data imply crosstalk between the macrophage-lineage cells and the CD4+ T cells, forming an amplification loop for the immune responses.

TAK is another major large-vessel type vasculitis. The histologic pathology includes granulomatous formation and accompanying deconstruction of the tunica media and intimal hyperplasia, as in GCA. The immune responses likely involve CD8+ T cells, whereas in GCA, it predominantly involves CD4+ T cells with the presence of CD8+ T cells [48, 49]. *MMP12*, *ATP6V0D2* and *LRRC15*, upregulated in TAK as well as GCA, may drive the tissue-remodelling of the affected arteries. TB is caused by mycobacterium infection and SA has an unknown aetiology, and both of them involve granulomas of lymph nodes rather than blood vessels. *MMP12*, *MRC1* and *HLA-DRA*, which play important roles in both TB and SA [50], are shared with GCA (Fig. 4C). The data imply similar features in pathogenesis of the diverse granulomas, including tissue remodelling and immune stimulation, despite the difference of the affected tissues.

Collectively, our data extended the list of functional and pathology-relevant molecules in the infiltrating macrophages and MNGCs. They imply that the macrophage-lineage cells contribute to GCA pathogenesis through the deconstruction of the tunica media due to macrophage elastase under the phagocytic regulatory program, promotion of myofibroblast proliferation to support intimal hyperplasia, and the activation of CD4+ T cells to form an amplification loop of immune responses. Overlap of the molecules identified in GCA with the ones in other granulomatous diseases indicated that the contribution of the macrophage-lineage cells to pathogenesis is not limited to GCA.

## Supplementary Material

keae710_Supplementary_Data

## Data Availability

The microarray data is available in GEO under accession GSE267979.
