# Supplementary material for: Tissue degrading and remodelling molecules in giant cell arteritis
Source: Rheumatology (Oxford). 2025 Jan 22;64(5):3095–103. doi: 10.1093/rheumatology/keae710 (PMC12048063; doi:10.1093/rheumatology/keae710)

Supplemental figure 1

Dendrogram (Euclid distance + Ward)

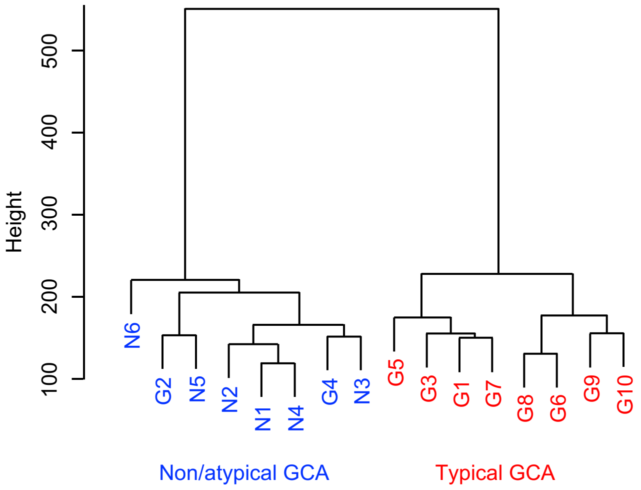

Supplemental figure 2

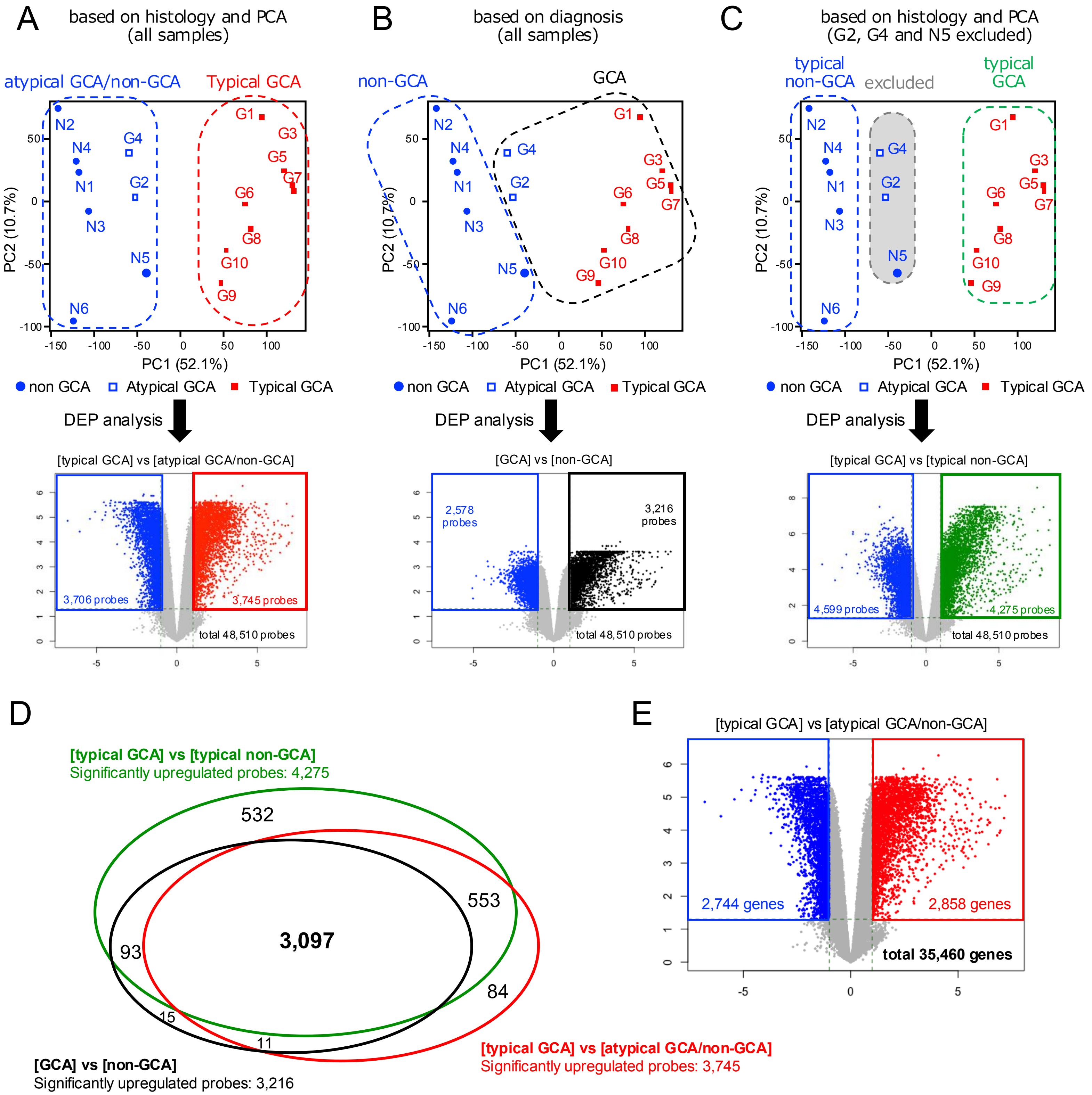

Supplemental figure 3

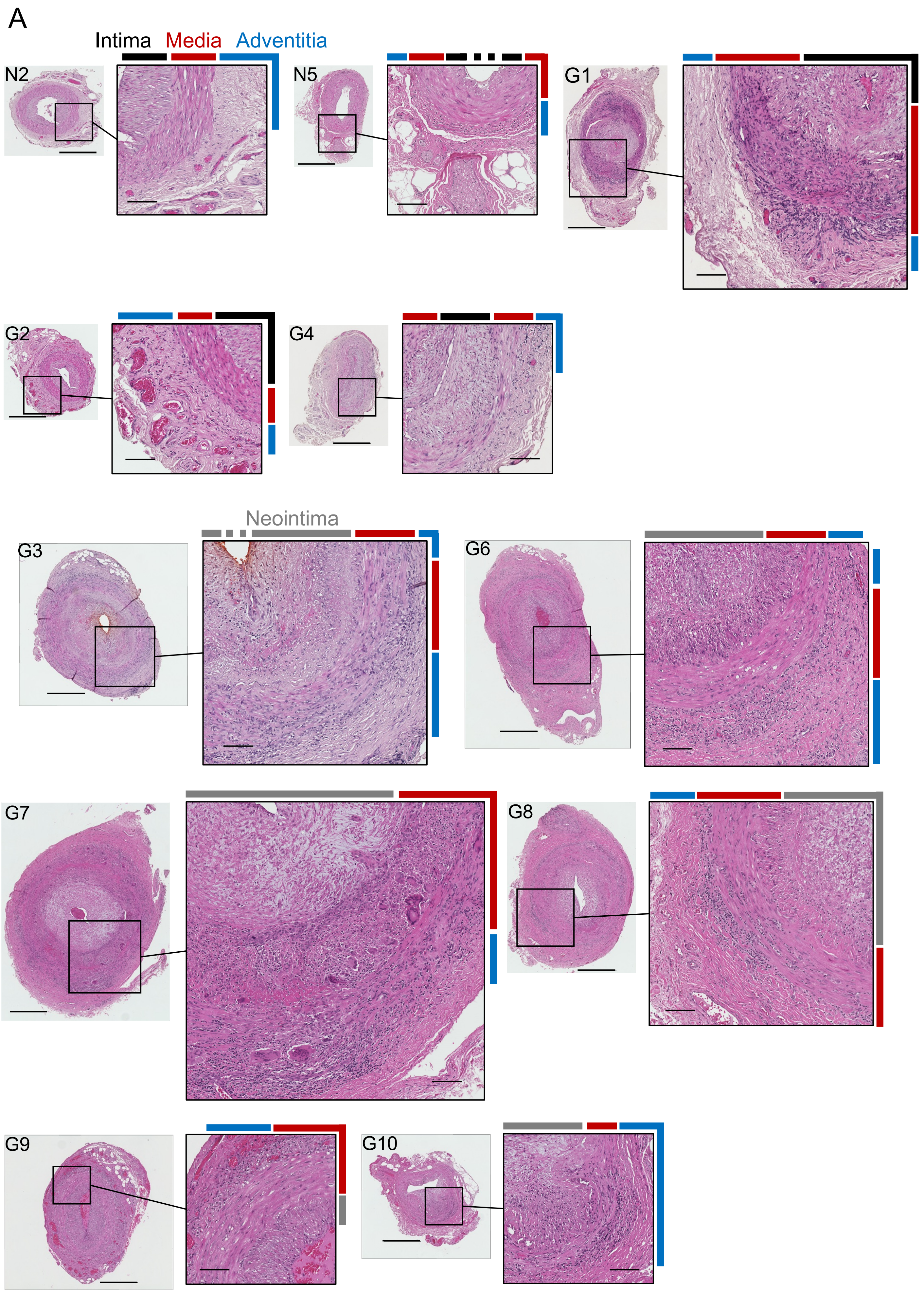

Supplemental figure 3

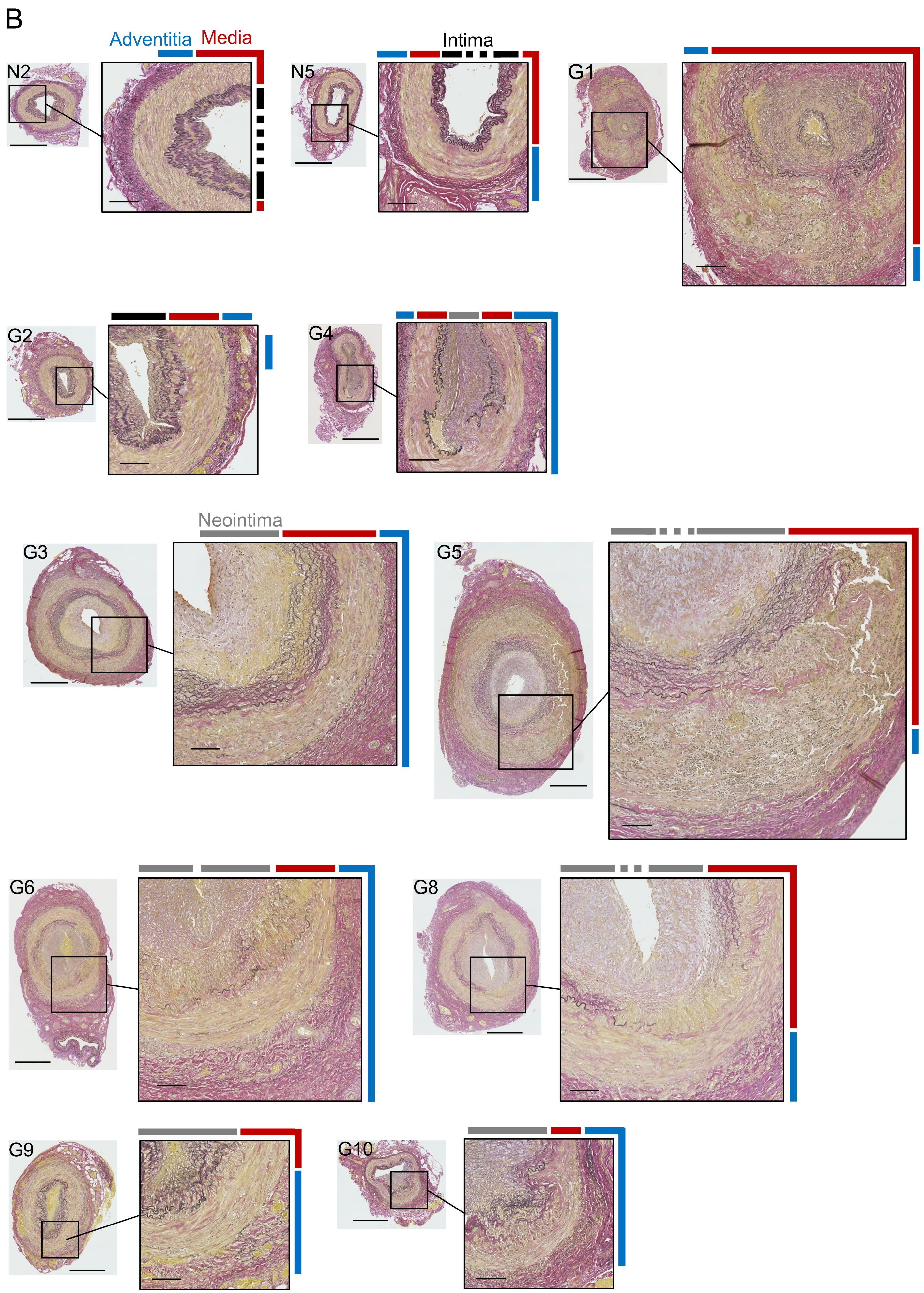

Supplemental figure 3

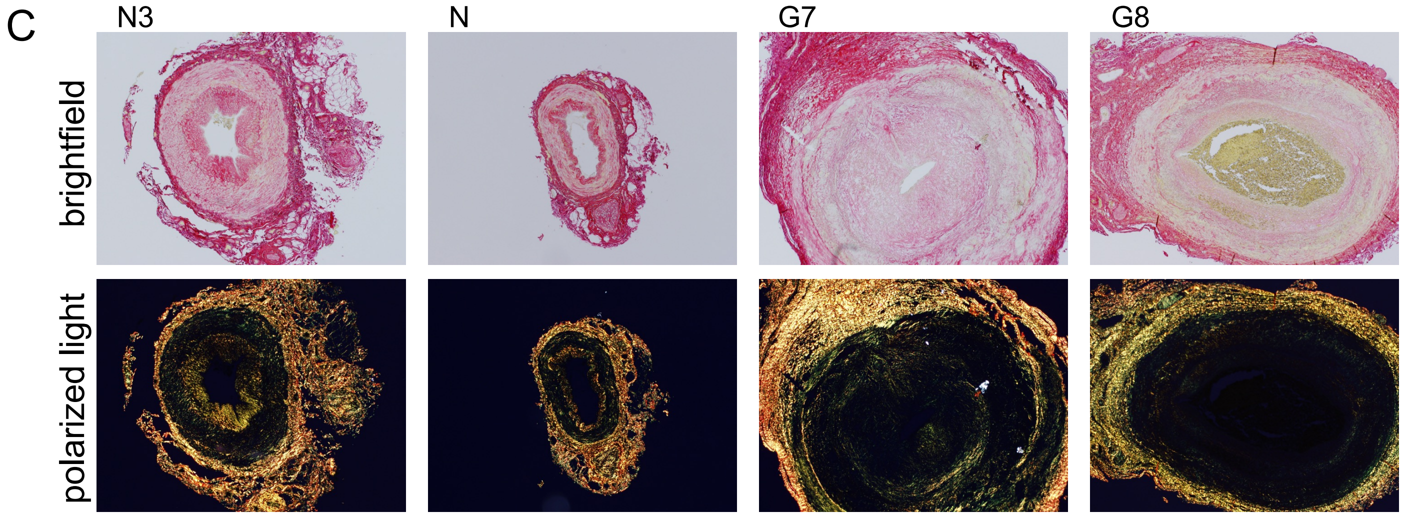

Supplementary figure 4

sample#	G7	G7	G7	G7
HIER	pH6	pH9	pH6	pH8
Ab	mouse IgG2b	mouse IgG2b	rabbit IgG	rabbit IgG
mg/mL	1	2	2	0.5
control for	ACP5	ATP6V0D2, CD206	VDR, HLA-DRA, TREM2, LRRC15	MMP12

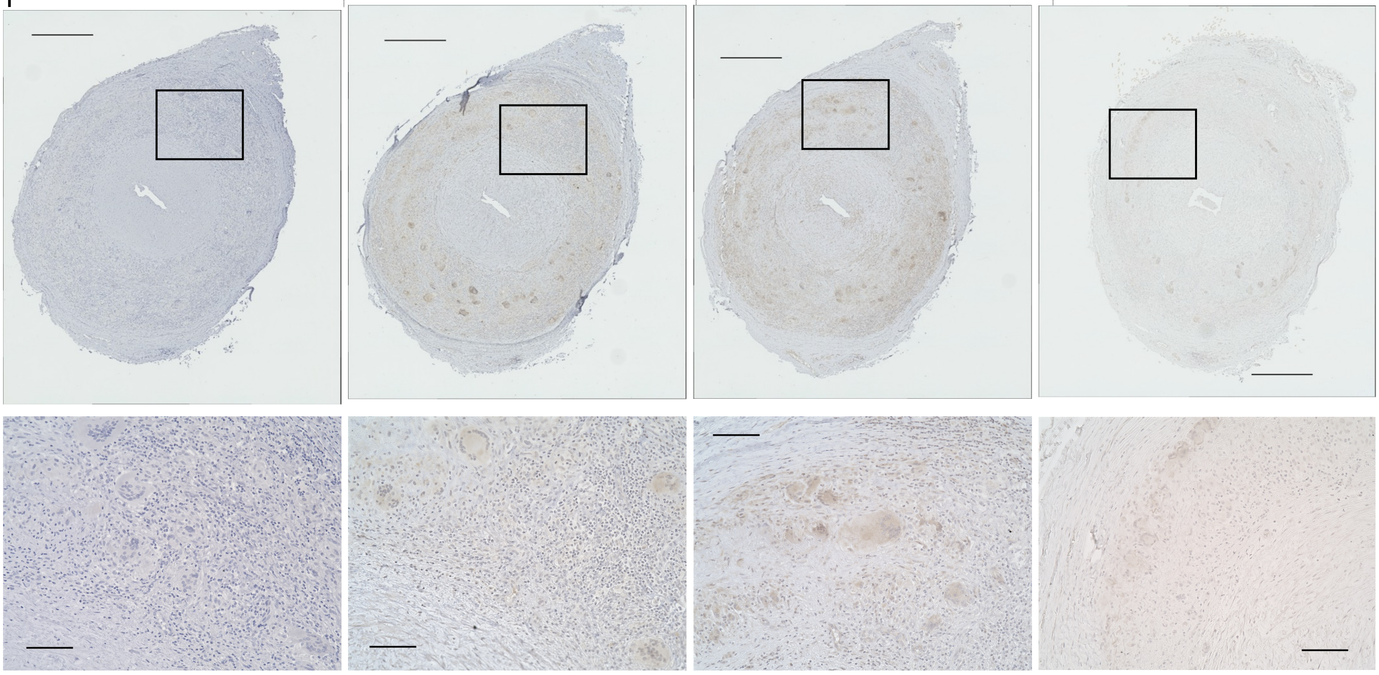

Supplemental figure 5

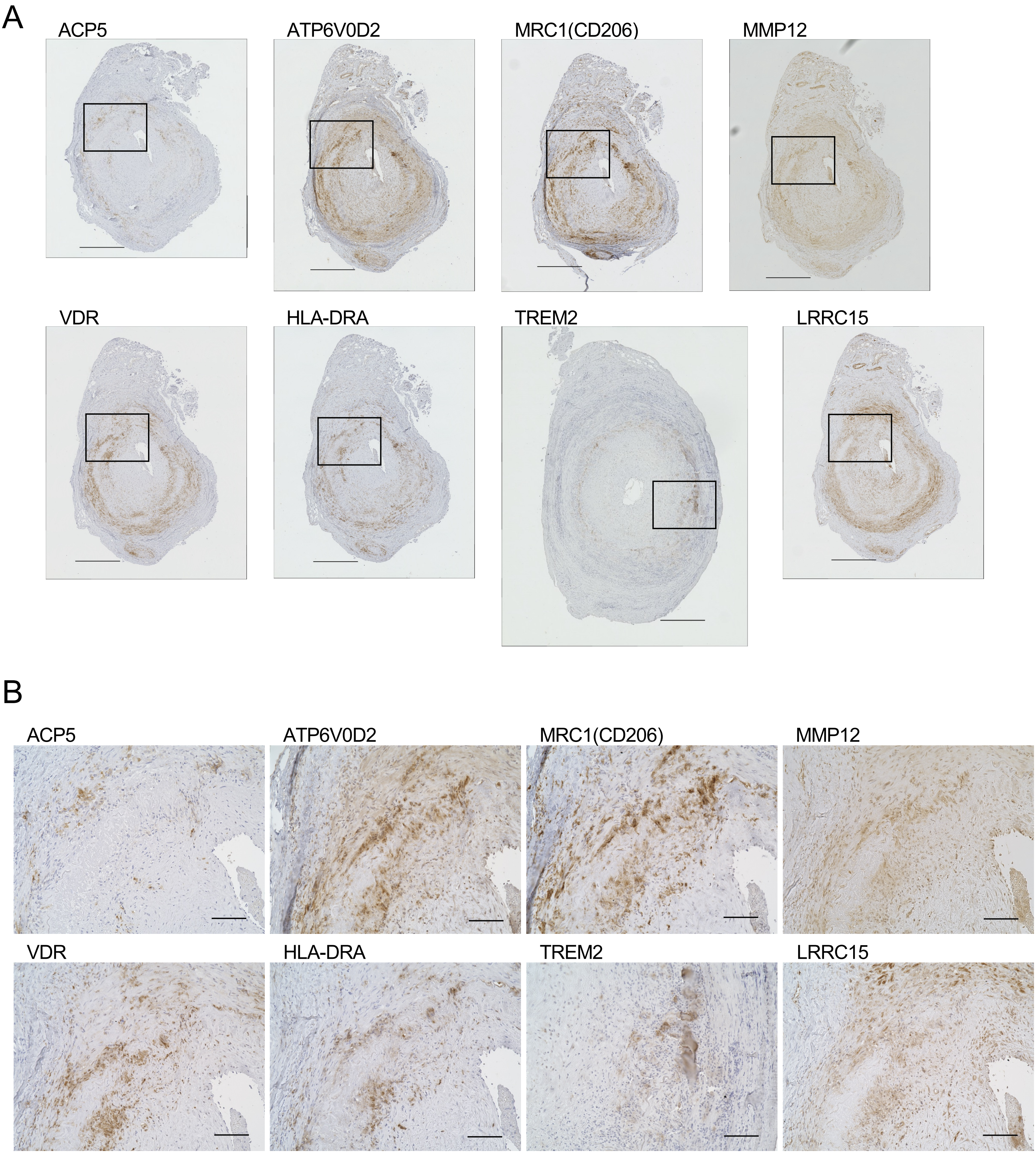

Supplemental figure 6

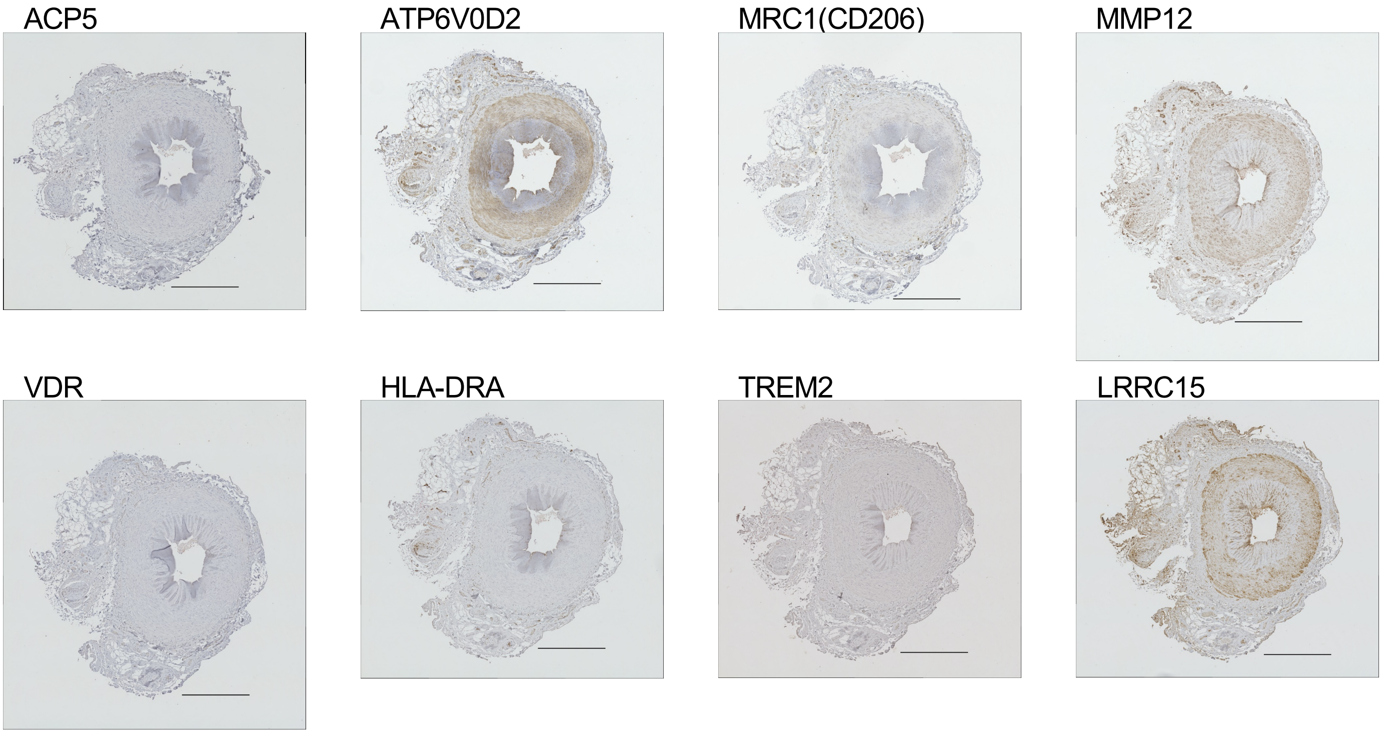

Supplemental figure 7

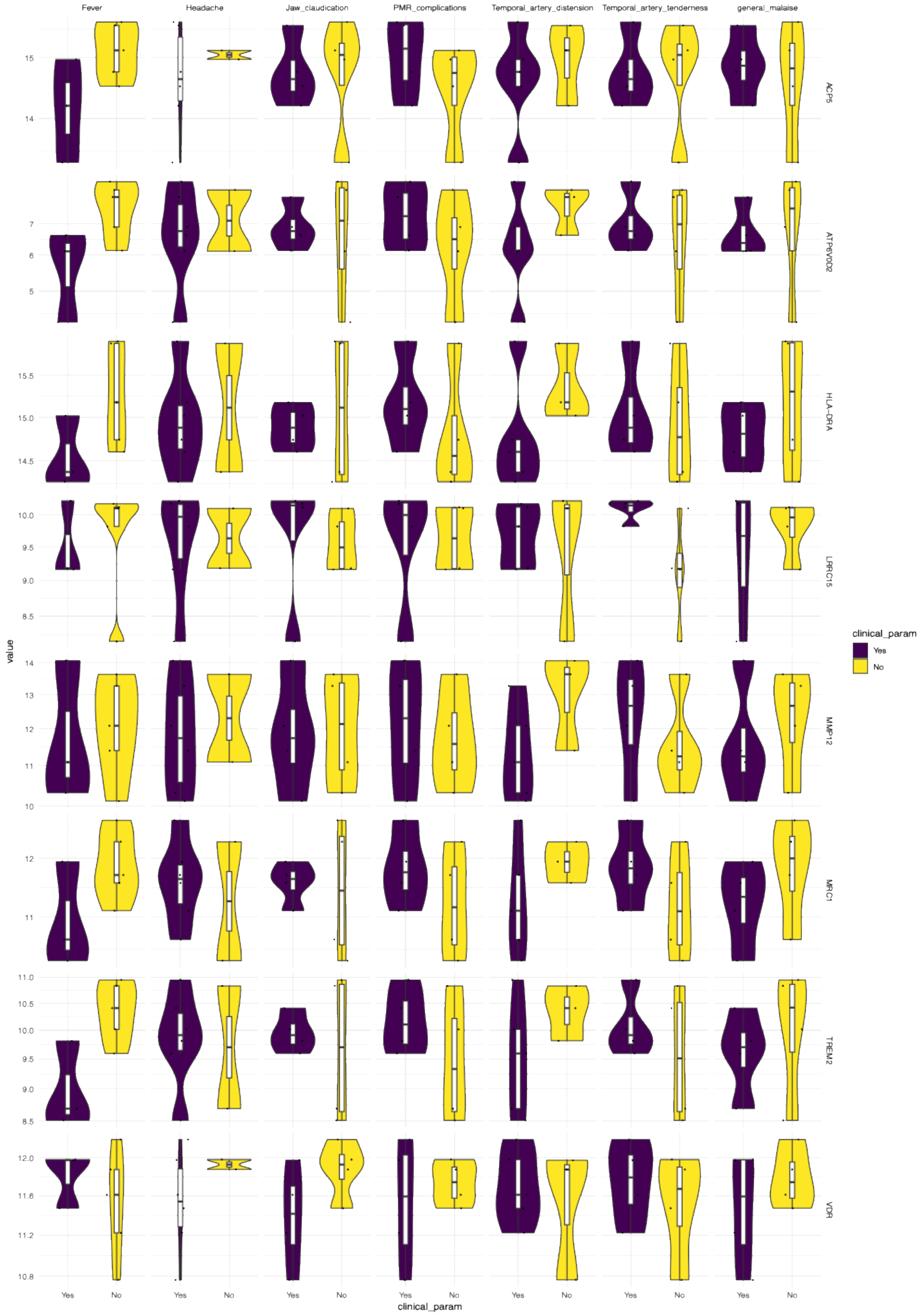

Supplement: keae710_Supplementary_Data [file keae710_supplementary_data.zip › keae710_Supplementary_Data/rhe-24-1565-File006.pdf]
