# Supplementary material for: Tissue degrading and remodelling molecules in giant cell arteritis
Source: Rheumatology (Oxford). 2025 Jan 22;64(5):3095–103. doi: 10.1093/rheumatology/keae710 (PMC12048063; doi:10.1093/rheumatology/keae710)
Supplement: keae710_Supplementary_Data [file keae710_supplementary_data.zip › keae710_Supplementary_Data/rhe-24-1565-File007.docx]

**SUPPLEMENTARY FIGURE LEGENDS**

**Supplementary Figure S1:** **Dendrogram obtained by Ward’s hierarchical clustering method using the Euclidean distance matrix.** GCA, giant cell arteritis.

**Supplementary Figure S2:** **Identification of differentially expressed probes (DEPs) and their conversion to differentially expressed genes (DEGs).**

(A) Classification of all samples based on both histology and PCA plots is shown on upper panel, and volcano plot comparing DEPs between the cluster for typical GCA and the atypical/non-GCA cluster shown on lower panel. Gray, no significant difference; red, DEPs significantly upregulated (log_2_FC > 1 and FDR < 0.05) in the typical GCA cluster; blue, DEPs significantly downregulated (log_2_FC < -1 and FDR < 0.05) in the typical GCA cluster.

(B) Classification of all samples based on diagnosis solely according to clinical findings is shown on upper panel, and volcano plot comparing DEPs between the GCA cluster and the non-GCA cluster shown on lower panel. Black, upregulated DEPs in the GCA cluster; blue, downregulated DEPs in the GCA cluster.

(C) After excluding G2, G4 and N5, which exhibit intermediate features, the classification based on histological analysis and PCA are shown on upper panel, and volcano plot comparing DEPs between the cluster for typical GCA and the typical non-GCA cluster shown on lower panel. Gray, no significant difference; red, DEPs significantly upregulated; blue, DEPs significantly downregulated in the typical GCA cluster versus tyical non-GCA cluster.

(D) Venn diagram showing the numbers of the overlapping upregulated DEPs between **A** (red), **B** (black) and **C** (green)

(E) Volcano plot comparing DEGs between the typical GCA cluster and non-GCA/atypical GCA cluster. A total of 48,510 probe sets were mapped to 35,460 genes according to the canonical manner described in Methods. Red, DEGs upregulated (log_2_FC > 1 and FDR < 0.05) in the typical GCA cluster; blue, DEGs downregulated (log_2_FC < -1 and FDR < 0.05) in the typical GCA cluster.

**Supplementary Figure S3:** **Histologic observation of representative temporal artery biopsies (TABs) used for the microarray analysis.**

(A) Representative images of hematoxylin-and-eosin–stained TABs derived from the same participants as in the microarray analysis. The entire arterial sections (scale bar: 500 μm) and high-magnification images (scale bar: 100 μm) of the boxed area of the entire arterial images. The layer structures of the artery, intima (neointima in typical GCA cases), tunica media, and adventitia are indicated.

(B) Representative images of TABs stained with Elastica van Gieson derived from the same participants as in the microarray analysis The entire arterial sections (scale bar: 500 μm) and high-magnification images (scale bar: 100 μm) of the boxed area of the entire arterial images. The layer structures of the artery, intima (neointima in typical GCA cases), tunica media, and adventitia are indicated.

(C) Images of representative non-GCA TABs (N3 and N5) and GCA TABs (G7 and G8) stained with picrosirius red to detect collagen subtypes. Upper panels, bright-field micrographs obtained under non-polarized light (scal bar: 500μm); lower panels, micrographs obtained under polarized light (scal bar: 500μm).

**Supplementary Figure S4:** **Staining with isotype controls.**

Formalin-fixed paraffin-embedded specimens of TABs from a representative patient with typical GCA (G7) were stained with the indicated control antibodies. The experimental conditions, buffer pH used for heat-induced epitope retrieval (HIER), antibody isotypes (Ab), and Ab concentrations are shown. Scale bars: upper panels, 500 μm; lower panels, 100 μm.

**Supplementary Figure S5:** **Immunohistochemistry of additional typical GCA samples.**

(A) Formalin-fixed paraffin-embedded specimens from representative patients with typical GCA (G6 and G8) were stained with the indicated antibodies. Scale bar: 500 μm.

(B) Magnifications of the areas corresponding to the boxed regions in **A**. Scale bar: 100 μm.

**Supplementary Figure S6:** **Immunohistochemistry of non-GCA samples.**

Formalin-fixed paraffin-embedded specimens from a representative patient with non-GCA (N3) were stained with the indicated antibodies. Scale bar: 500 μm

**Supplementary Figure S7: Expression levels of the identified marker genes depending on the clinical parameters described in Supplementary Table S1.**
